# Long-Term Effect of Bariatric Surgery on Liver Enzymes in the Swedish Obese Subjects (SOS) Study

**DOI:** 10.1371/journal.pone.0060495

**Published:** 2013-03-26

**Authors:** Maria Antonella Burza, Stefano Romeo, Anna Kotronen, Per-Arne Svensson, Kajsa Sjöholm, Jarl S. Torgerson, Anna-Karin Lindroos, Lars Sjöström, Lena M. S. Carlsson, Markku Peltonen

**Affiliations:** 1 Department of Molecular and Clinical Medicine and Center for Cardiovascular and Metabolic Research, Sahlgrenska Academy, University of Gothenburg, Gothenburg, Sweden; 2 Clinical Nutrition Unit, Department of Medical and Surgical Sciences, University Magna Graecia, Catanzaro, Italy; 3 Department of Chronic Disease Prevention, National Institute for Health and Welfare, Helsinki, Finland; 4 Department of Health Care, Regional Secretariat, Västra Götaland Region, Gothenburg, Sweden; 5 Nutrition Department, National Food Administration, Uppsala, Sweden; University of Verona, Ospedale Civile Maggiore, Italy

## Abstract

**Background and Aim:**

Obesity is associated with elevated serum transaminase levels and non-alcoholic fatty liver disease and weight loss is a recommended therapeutic strategy. Bariatric surgery is effective in obtaining and maintaining weight loss. Aim of the present study was to examine the long-term effects of bariatric surgery on transaminase levels in obese individuals.

**Methods:**

The Swedish Obese Subjects (SOS) study is a prospective controlled intervention study designed to compare the long-term effects of bariatric surgery and usual care in obese subjects. A total of 3,570 obese participants with no excess of alcohol consumption at baseline (1,795 and 1,775 in the control and surgery group, respectively) were included in the analyses. Changes in transaminase levels during follow-up were compared in the surgery and control groups.

**Results:**

Compared to usual care, bariatric surgery was associated with lower serum ALT and AST levels at 2- and 10- year follow up. The reduction in ALT levels was proportional to the degree of weight loss. Both the incidence of and the remission from high transaminase levels were more favorable in the surgery group compared to the control group. Similarly, the prevalence of ALT/AST ratio <1 was lower in the surgery compared to the control group at both 2- and 10-year follow up.

**Conclusions:**

Bariatric surgery results in a sustained reduction in transaminase levels and a long-term benefit in obese individuals.

## Introduction

Elevated serum transaminase levels are frequently associated with obesity [1]–[3] and with progression to chronic liver disease [4], [5], including non-alcoholic liver disease (NAFLD). Bariatric surgery is the most effective strategy to obtain and maintain weight loss in obese individuals [6]. Results from uncontrolled studies [7]–[13] and two small controlled studies [14], [15] indicate that weight loss obtained by bariatric surgery reduces transaminases and NAFLD. Previous studies have shown the favorable effect of weight loss obtained by bariatric surgery on transaminase levels, however, data from large controlled studies with long-term follow up, are not available [16]. The aim of the current report was to examine the long-term effects of bariatric surgery on serum transaminases in the prospective, controlled study, the Swedish obese subjects (SOS) study.

## Materials and Methods

### The Swedish Obese Subjects (SOS) Study

The SOS study is a non-randomized, matched, prospective, controlled, intervention trial that compares the long-term effects of bariatric surgery and usual care in obese subjects. The SOS study and its inclusion and exclusion criteria have been previously described [17] and they were identical for the two treatment groups. Briefly, from September 1^st^ 1987 to January 31^st^ 2001, a total of 4,047 obese individuals were recruited: 2,010 individuals who selected surgical treatment constituted the bariatric surgery group and a matched control group of 2,037 individuals was enrolled based on 18 matching variables.

Individuals in the surgery group underwent either nonadjustable or adjustable banding (n = 376), vertical banded gastroplasty (n = 1369) or gastric bypass (n = 265). Subjects in the control group received the conventional treatment for obesity at their center of registration (advanced lifestyle modification, other or no treatment). Inclusion criteria were age between 37 to 60 years and BMI of ≥34 kg/m^2^ for men and ≥38 kg/m^2^ for women. The exclusion criteria were: earlier bariatric surgery, earlier surgery for gastric or duodenal ulcer, gastric ulcer during the past six months, ongoing malignancy, active malignancy during the past five years, myocardial infarction during the past six months, bulimic eating pattern, psychiatric or cooperative problems contraindicating bariatric surgery, regular use of cortisone or non-steroidal anti-inflammatory treatment; excess of alcohol intake and alcohol or drug abuse, and other severe illnesses. The current and past health status was assessed by extensive questionnaires, completed by patients, and subsequently by health examination. The daily alcohol consumption was collected from the validated SOS dietary questionnaire [18].

The baseline examination took place approximately four weeks before the date of bariatric surgery for both the surgery patients and the matched control individuals. At baseline examination and after 2 and 10 years, anthropometric, clinical and biochemical parameters were measured. Blood samples were obtained in the morning after an overnight fast and analyzed at the Central Laboratory of Sahlgrenska University Hospital (accredited according to European Norm 45001).

In the current report, changes in serum transaminase levels and body weight were calculated as the difference between follow up (2 or 10 years) and baseline values. In addition, incidence of high transaminase during follow-up, as well as remission from high transaminase at baseline were analyzed. Specifically, the high transaminase group was defined by AST levels ≥33 U/L or 29 U/L and ALT levels ≥43 U/L or 30 U/L in men or women, respectively, as cut-offs. These transaminase cut-off levels have been shown to define NAFLD, indicated as liver fat content >5.6% by proton magnetic resonance spectroscopy in individuals with alcohol intake ≤20 (men) or ≤10 g/day (women) [19]. To be consistent with this previous report, SOS study participants with baseline self-reported alcohol consumptions of >20 g/day (men) or >10 g/day (women) were not included (n = 427) in the current analyses. Furthermore, individuals lacking data on baseline AST and ALT levels (n = 50) were also excluded from the analyses resulting in a total of 3,570 subjects examined in the current report. The ALT/AST ratio was calculated in the follow up as an index of severe liver damage [20]. Follow-up data were available for 3,102 (87%) persons after 2 years, and for 2,157 (60%) after 10 years. The cut-off date for our current analysis was March 20^th^, 2009.

### Ethics Statement

SOS trial has been registered in the ClinicalTrials.gov registry (NCT01479452, http://clinicaltrials.gov/ct2/show/NCT01479452?term=NCT01479452&rank=1). Written informed consent has been obtained by all the study participants. The study was approved by all the relevant ethics review boards in Sweden.

### Statistical Analyses

Characteristics of the study population are showed as number and percentage for categorical variables or as means and standard deviations (SD) for continuous variables. Changes in anthropometric and laboratory variables between the treatment groups were compared with analysis of covariance (ANCOVA), adjusting for age, gender, baseline BMI and the baseline value of the respective variable. Results are presented as mean difference in changes between the surgery and control groups, together with corresponding 95% confidence intervals. Spearman rank correlation tests were performed to determine the relationship between transaminase and weight changes. For incidence and remission calculations, logistic regression models were used, adjusting for age, gender and baseline BMI. The prevalence of the ALT/AST ratio <1 in the control and in the surgery group was compared by Fisher’s exact test. Analyses were performed with the statistics package Stata version 9.2 [21].

## Results

### Baseline Characteristics

A total of 3,570 individuals (control group, n = 1,795; surgery group, n = 1,775) from the overall SOS study population were included in the current analyses. Baseline characteristics of the surgery and control groups are described in Table 1. Individuals in the surgery group were younger than individuals in the control group. Furthermore, the surgery group had higher body weight, BMI, blood pressure, glucose and liver transaminase levels compared to the control group. No differences in other parameters including alcohol consumption and use of lipid- and blood glucose-lowering medications were observed in the two treatment groups. For incidence and remission calculations, the SOS individuals were stratified in groups with low (LT) and high serum transaminase (HT) levels. The baseline characteristics of these subgroups are shown in Table S1. At baseline, the prevalence of HT levels was 46% (n = 818) in the surgery group and 36% (n = 645) in the control group (P value<0.001). In both groups, individuals in the surgery group were younger and had higher body weight, BMI, blood pressure, glucose compared to the control group, reflecting the overall population characteristics (Table 1).

**Table 1:** Baseline characteristics of the individuals from the SOS study included in this report.

	Control	Surgery	P value
**N**	1795	1775	
**Gender F, %**	72	73	0.82
**Age, years**	49±6	47±6	<0.001
**Body weight, kg**	115±16	121±17	<0.001
**BMI, kg/m^2^**	40.2±4.7	42.4±4.5	<0.001
**Systolic blood pressure, mmHg**	138±18	145±19	<0.001
**Diastolic blood pressure, mmHg**	85±11	90±11	<0.001
**Blood glucose, mmol/L**	4.9±1.8	5.2±2.0	<0.001
**Total cholesterol, mmol/L**	5.6±1.1	5.9±1.1	<0.001
**Triglycerides, mmol/L**	1.99±1.34	2.24±1.58	<0.001
**HDL cholesterol, mmol/L**	1.34±0.33	1.34±0.32	0.92
**ALT, U/L**	33±26	37±23	<0.001
**AST, U/l**	23±12	25±14	<0.001
**Alcohol consumption,** **g/day**	3.2±4.3	3.3±4.2	0.38
**Diabetes, %**	13	18	<0.001
**Glucose-lowering** **medication, %**	7	8	0.06
**Lipid-lowering** **medication, %**	2	2	0.44

Data are shown as mean ± SD, or proportion.

*Abbreviations*: SOS, Swedish obese subjects; N, number; F, female; BMI, body mass index; HDL, high density lipoprotein; ALT, alanine transferase; AST, aspartate transferase.

### Changes in Serum Transaminase Levels and Body Weight

Bariatric surgery was associated with a significant and sustained decrease in body weight compared to the control group [6].

**Figure 1:**
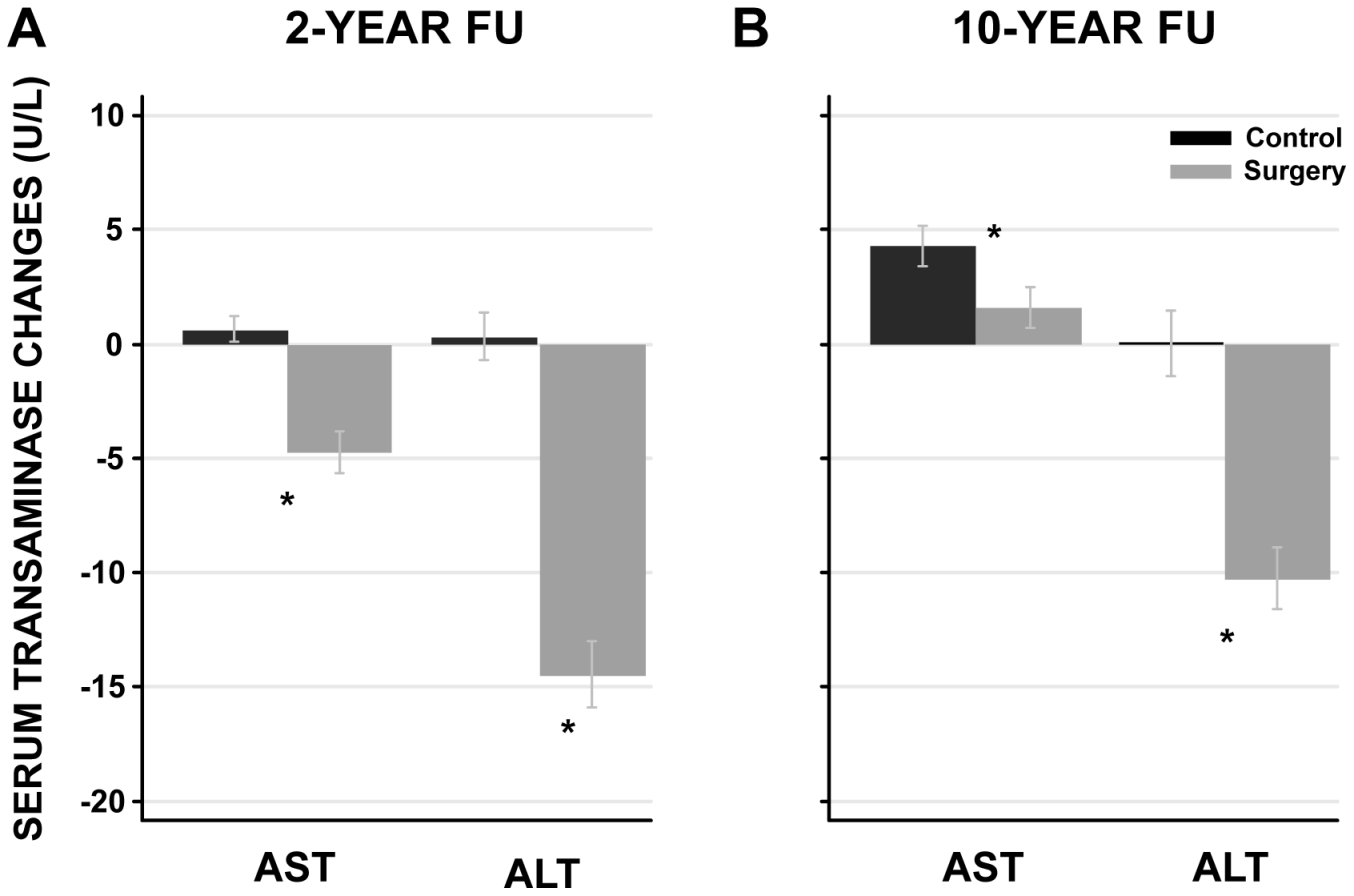
Serum transaminase changes in the surgery and control groups at 2- and 10-year follow up. The serum transaminase changes are expressed as mean and 95% confidence intervals. Changes are calculated as the difference between follow up (2 or 10 year) and baseline values. *P value <0.001, surgery vs. control group. *Abbreviations:* FU, follow up; AST, aspartate transferase; ALT, alanine transferase.

**Figure 2:**
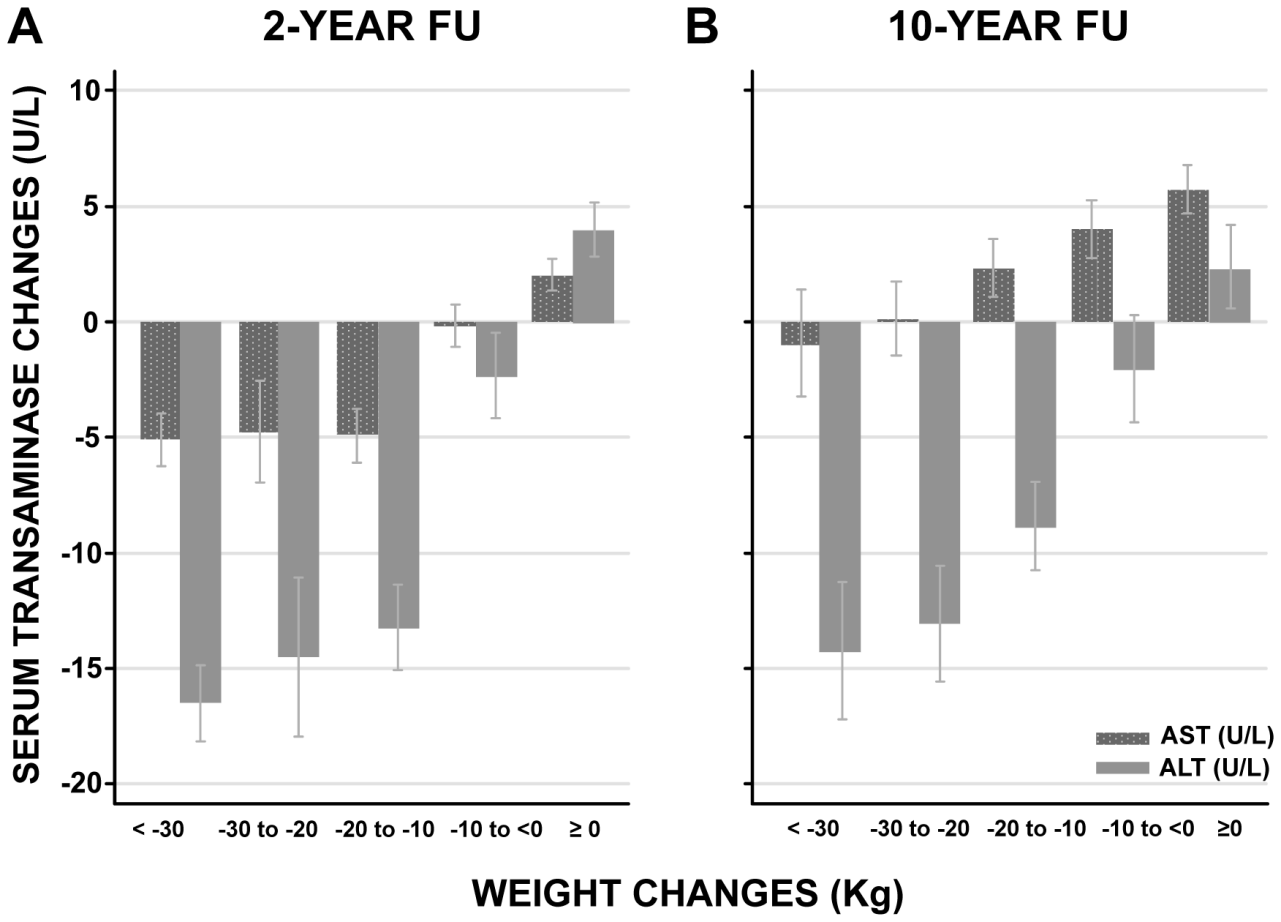
Serum transaminase changes and their relation to body weight changes at 2- and 10-year follow up. The serum transaminase changes are expressed as mean and 95% confidence intervals. The surgery and control groups are pooled. Changes are calculated as the difference between follow up (2 or 10 year) and baseline values. *Abbreviations:* FU, follow up; AST, aspartate transferase; ALT, alanine transferase.

After two years, both ALT and AST were reduced in the surgery group, while there was no change in the control group (Figure 1A). After ten years, this pattern remained for ALT, while there was an increase in AST, which was significantly smaller in the surgery group than in the control group (Figure 1B). Moreover, serum transaminase level changes were positively correlated to body weight changes at both 2- (ALT: r = 0.500, P value<0.001; AST: r = 0.289, P value<0.001) and 10- year (ALT: r = 0.357, P value<0.001; AST: r = 0.160, P value<0.001) follow up when the surgery and control groups were pooled (Figure 2A and B; Figure S1 and S2). There was a continuous reduction in ALT levels with increasing weight loss for both the 2- and 10-year follow-up. Similarly, serum AST level were related to weight loss, however, there was no further reduction in AST levels beyond weight loss of more than 10 kg at 2-year follow-up (Figure 2A). At 10-year follow up, the AST levels were associated with weight change, but compared to baseline there was no reduction in the AST levels irrespective of the weight change categories (Figure 2B). Weight gain was associated with increased serum transaminases at both 2- and 10-year follow-up (Figure 2A and B).

### Incidence of and Remission from HT

**Figure 3:**
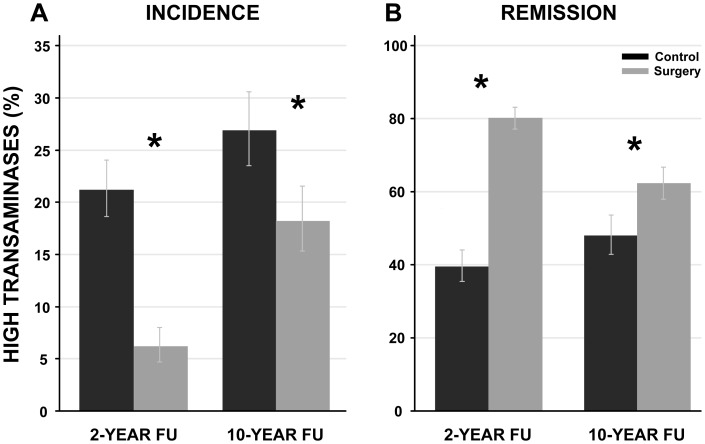
High transaminase (HT) incidence and remission in the surgery and the control groups at 2- and 10-year follow up. HT group was defined if one or both transaminase levels were above the following cut-offs: AST ≥33 U/L (men) and 29 U/L (women) ALT levels ≥43 U/L (men) and 30 U/L (women). Incidence and remission are expressed as proportion of individuals; the bars indicate the 95% confidence intervals. *P value <0.001, surgery vs. control group. A. Control n = 916 and 621 at 2- and 10-year FU; Surgery n = 857 and 618 at 2- and 10 -year FU. B. Control n = 519 and 332 at 2- and 10-year FU; Surgery n = 731 and 491 at 2- and 10-year FU. *Abbreviations:* FU, follow up; HT, high transaminase; AST, aspartate transferase; ALT, alanine transferase.

The incidence of HT was lower in the surgery compared to the control group at both 2-year (6% and 21%, for surgery and control groups, respectively P value<0.001) and at 10-year (18% and 27%, p<0.001) follow up (Figure 3A). A reduced risk for HT onset was observed in the surgery group at 2 and 10 years (OR: 0.26 (95% confidence interval 0.18–0.36, P value<0.001) and 0.61 (95% confidence interval 0.46–0.81, P value<0.001, respectively). Moreover, remission from HT was more common in the surgery group compared to the control group (80% and 39%, for surgery and control groups, respectively after 2-years, P value<0.001, and 63% and 47%, respectively after 10-years, P value<0.001) (Figure 3B). An increased probability for HT remission was observed at 2- and 10- year follow up in the surgery group (OR: 6.55 (95% CI 5.0–8.5) and 1.80 (95% CI 1.35–2.40) at 2- and 10-year follow up, both P values<0.001).

**Figure 4:**
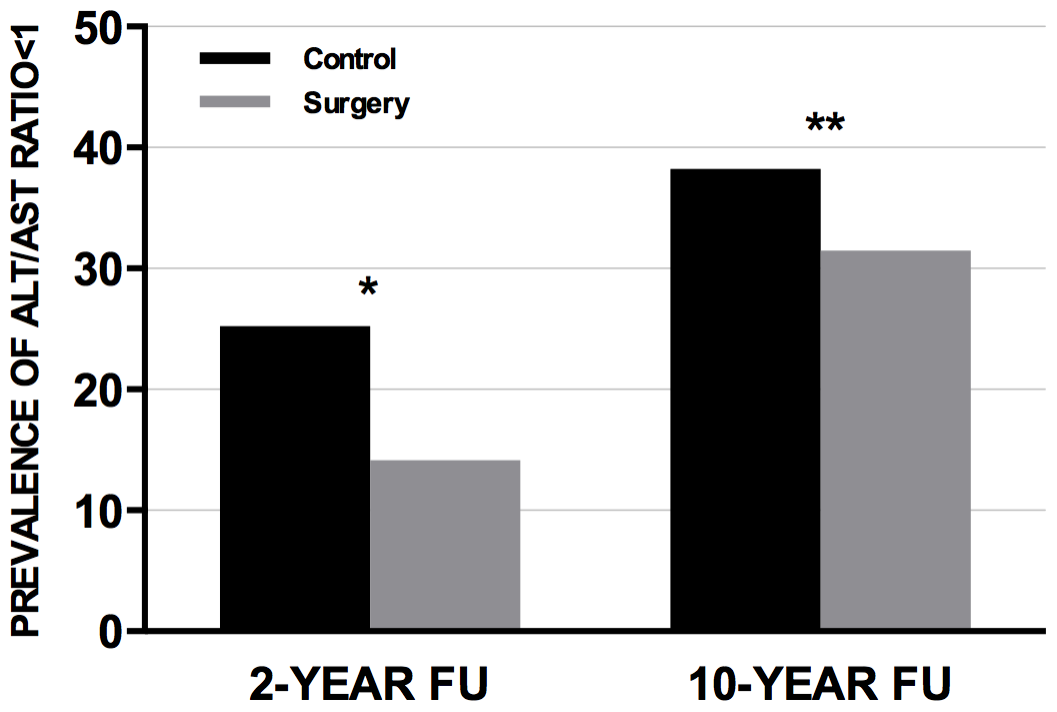
Prevalence of ALT/AST ratio <1 in the surgery and the control groups at 2- and 10-year follow up. The prevalence of ALT/AST ratio <1 is expressed as proportion of individuals. The differences in the prevalence of ALT/AST ratio<1 between the control and the surgery group has been analyzed by Fisher’s exact test. Prevalence of ALT/AST ratio <1 was: 25% (n = 368, control group) and 14% (n = 231, surgery group) at 2-year follow-up; 38% (n = 381, control group) and 31% (n = 366, surgery group) at 10-year follow-up. *P value <0.001, surgery vs. control group. **P value = 0.001, surgery vs. control group. *Abbreviations*: ALT, alanine transferase; AST, aspartate transferase; FU, follow up.

Finally, the proportion of individuals with an ALT/AST ratio <1 was calculated in the control and in the surgery group in the follow-up (Figure 4). The prevalence of ALT/AST ratio <1 was lower in the surgery compared to the control group at both 2-year (14% vs. 25% respectively, P value <0.001) and 10-year follow up (31 vs. 38% respectively, P value = 0.001).

## Discussion

Elevated serum transaminase levels are commonly associated with obesity [1]–[3] and with progression to chronic liver disease. Bariatric surgery is the most effective strategy to achieve and maintain weight loss [6]. In the present report, the long-term effect of bariatric surgery on serum transaminases has been examined.

A sustained reduction up to 10 years of both ALT and AST levels was observed in the surgery compared to the control group. Several studies investigating the effect of bariatric surgery on liver enzymes and NAFLD have shown an improvement of serum transaminases and hepatic histologic features after surgery [7]–[15]. The novelty of the current report is the observation of a beneficial effect of bariatric surgery on serum transaminases that is maintained up to 10 years after surgery. These data suggest that bariatric surgery has a long-term protective effect against chronic liver damage. The effects after 10 years were smaller than after 2 years and this is likely due to the weight regain observed 10 years after bariatric surgery [22]. Furthermore, analysis of the relation between changes in transaminase levels and changes in body weight showed that weight gain was associated with a significant increase of transaminase levels. ALT showed a continuous linear reduction with increasing weight loss at the 2-year follow up which was maintained at the 10 year follow up. These data indicate that sustained weight loss has a beneficial long-term effect on chronic liver damage and that this effect is proportional to the weight loss reduction.

When changes in AST levels were examined, a reduction related to weight loss was observed at the 2-year but not at the 10-year follow up. The reason for this unexpected finding is unknown but it may be due to factors that have changed over time (e.g., age, environmental factors, lifestyle habits, medications) and influenced transaminase differently [23]. Nonetheless, it is worth noting that despite the absence of reduction at 10-year follow up, the AST levels remained lower in the surgery when compared to the control group.

Furthermore, the long-term effect of bariatric surgery on HT incidence and remission was examined. The incidence of and the remission from HT at both 2- and 10-year follow up were significantly more favorable in the surgery group compared to the control group. Similarly, the prevalence of an ALT/AST ratio <1, an index of severe liver disease [20], was lower in the surgery group compared to the control group at both 2- and 10-year follow up.

Taken as a whole, these data suggest that weight reduction has a positive effect on liver transaminases and chronic liver damage.

Strength of the present report is that we examined the effect of bariatric surgery on liver transaminase in a large obese cohort with a long-term follow up. Previous reports with different study design and shorter follow up have shown that both weight loss induced by lifestyle intervention and bariatric surgery reduce serum transaminase levels and NAFLD [7]–[15]. Our data confirm these short-term effects in a matched, controlled, prospective study including more than 3,000 obese individuals and also show that the effects are sustained during a 10-year follow up. A limitation of the present report is that the effect of bariatric surgery on transaminase levels was not a predefined endpoint of the SOS study, although the transaminase level measurements were performed prospectively. Thus, our analyses should be considered exploratory. Another limitation is that even though no clinical diagnosis of advanced chronic liver disease was present, we cannot exclude the presence of relevant liver injury at baseline.

Taken all this together it may be speculated that sustained weight loss obtained by bariatric surgery reduces liver damage and may possibly prevent hepatic long-term sequelae. Further longitudinal studies, using more sensitive techniques to assess chronic liver disease, are warranted to confirm these data. In conclusion, this report shows that bariatric surgery is associated with long-term reduction of serum transaminases in obese individuals.

## Supporting Information

Figure S1 **Association between weight and ALT level changes at 2- and 10-year follow up.** Changes are calculated as the difference between follow up (2 or 10 years) and baseline values. The surgery and control groups are pooled. Spearman rank correlation tests were performed to determine the relationship between transaminase and weight changes. ALT level changes correlate to body weight changes at both 2- (ALT: r = 0.500, P value<0.001) and 10-year (ALT: r = 0.357, P value<0.001) follow up. *Abbreviations:* ALT, alanine transferase.
(TIF)

Figure S2 **Association between weight and AST level changes at 2- and 10-year follow up.** Changes are calculated as the difference between follow up (2 or 10 years) and baseline values. The surgery and control groups are pooled. Spearman rank correlation tests were performed to determine the relationship between transaminase and weight changes. AST level changes correlate to body weight changes at both 2- (AST: r = 0.289, P value<0.001) and 10-year (AST: r = 0.160, P value<0.001) follow up. *Abbreviations:* AST, aspartate transferase.
(TIF)

Table S1 **Baseline characteristics of SOS individuals with LT and HT.** Data are shown as mean ± SD, or proportions. *Abbreviations*: SOS, Swedish obese subjects; LT, low transaminase; HT, high transaminase; N, number; F, female; BMI, body mass index; HDL, high density lipoprotein; ALT, alanine transferase; AST, aspartate transferase.
(DOC)
