# Supplementary figures and images for: Long-Term Effect of Bariatric Surgery on Liver Enzymes in the Swedish Obese Subjects (SOS) Study
Source: PLoS One. 2013 Mar 26;8(3):e60495. doi: 10.1371/journal.pone.0060495 (PMC3608624; doi:10.1371/journal.pone.0060495)

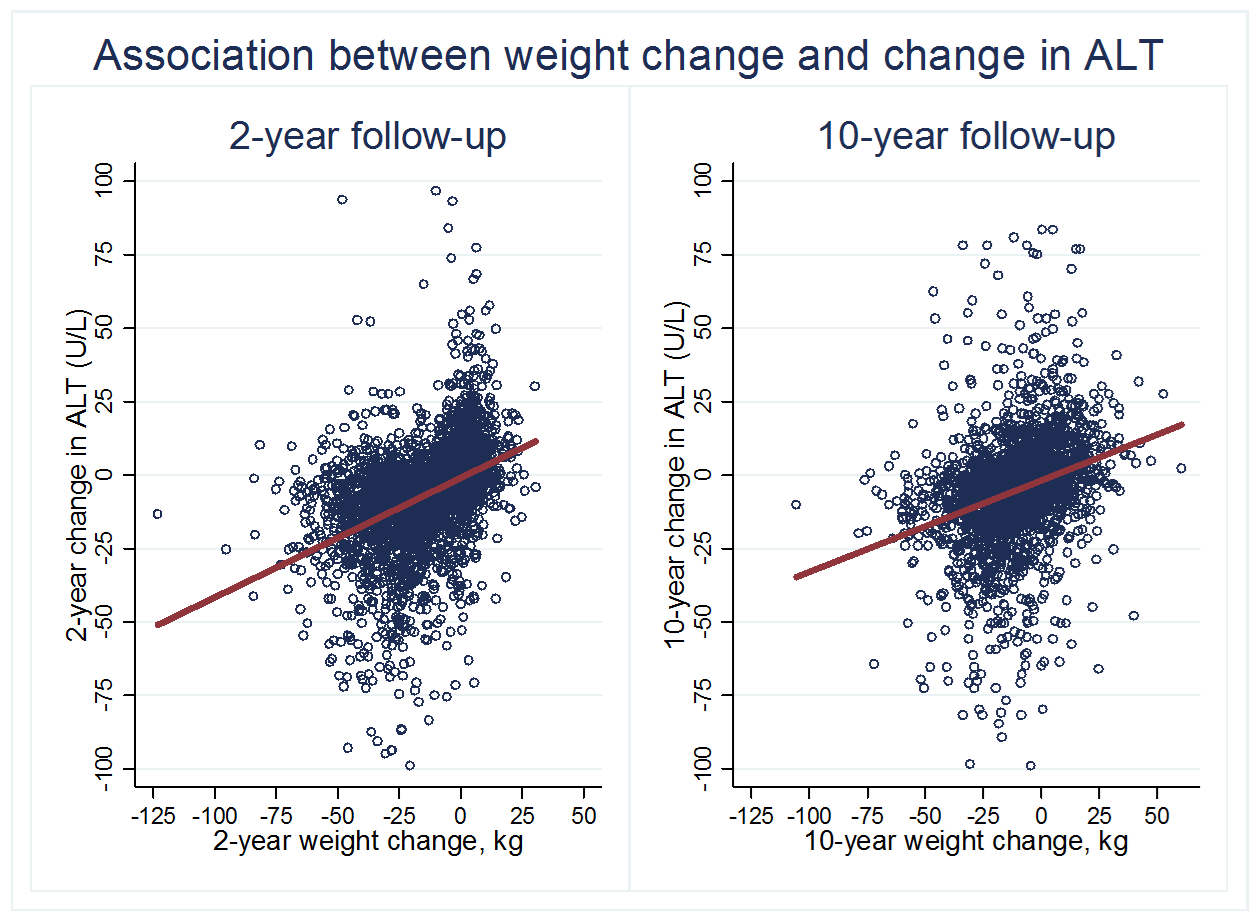

Supplement: Figure S1 — Association between weight and ALT level changes at 2- and 10-year follow up. Changes are calculated as the difference between follow up (2 or 10 years) and baseline values. The surgery and control groups are pooled. Spearman rank correlation tests were performed to determine the relationship between transaminase and weight changes. ALT level changes correlate to body weight changes at both 2- (ALT: r = 0.500, P value<0.001) and 10-year (ALT: r = 0.357, P value<0.001) follow up. Abbreviations: ALT, alanine transferase. (TIF) [file pone.0060495.s001.tif]

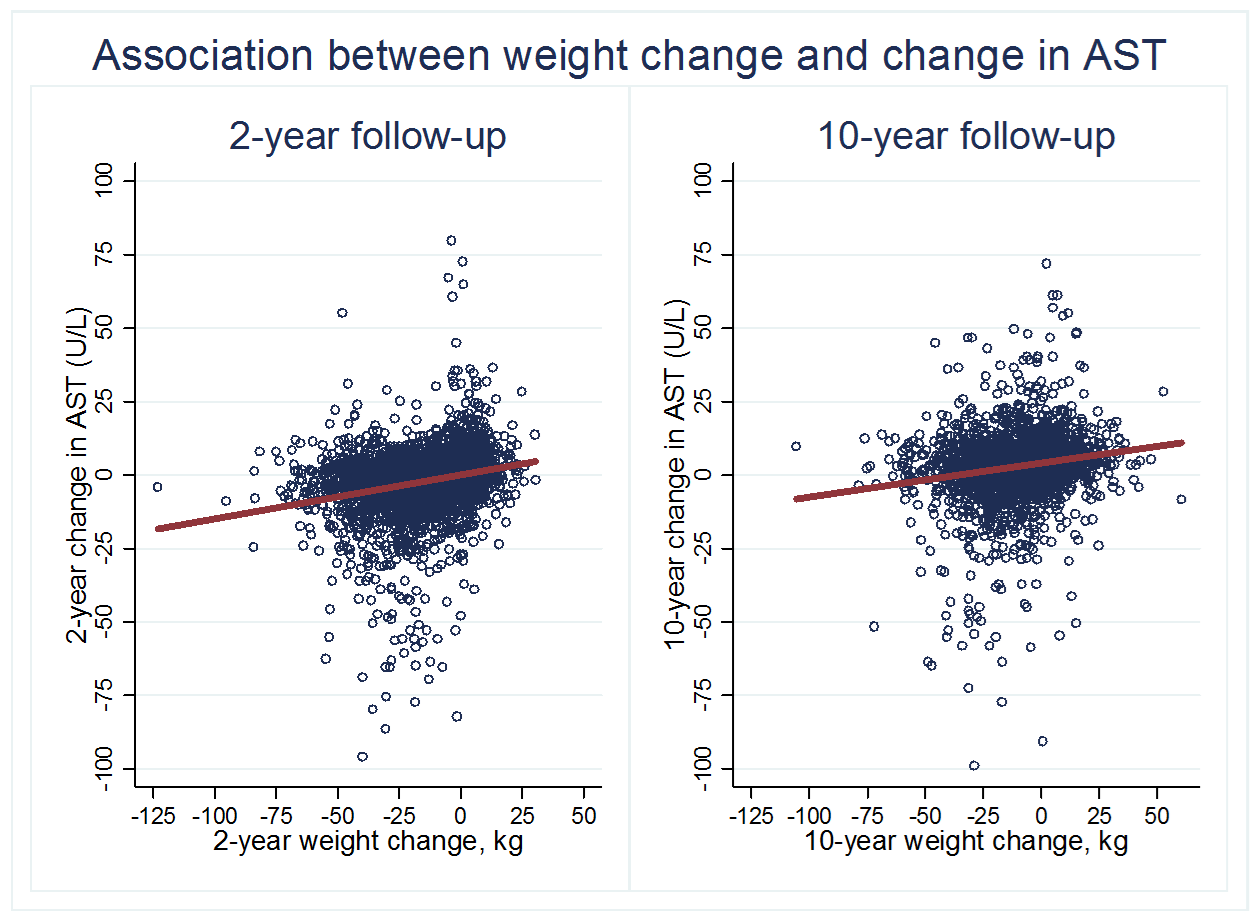

Supplement: Figure S2 — Association between weight and AST level changes at 2- and 10-year follow up. Changes are calculated as the difference between follow up (2 or 10 years) and baseline values. The surgery and control groups are pooled. Spearman rank correlation tests were performed to determine the relationship between transaminase and weight changes. AST level changes correlate to body weight changes at both 2- (AST: r = 0.289, P value<0.001) and 10-year (AST: r = 0.160, P value<0.001) follow up. Abbreviations: AST, aspartate transferase. (TIF) [file pone.0060495.s002.tif]
