# Supplementary material for: Long-Term Effect of Bariatric Surgery on Liver Enzymes in the Swedish Obese Subjects (SOS) Study
Source: PLoS One. 2013 Mar 26;8(3):e60495. doi: 10.1371/journal.pone.0060495 (PMC3608624; doi:10.1371/journal.pone.0060495)
Supplement: Table S1 — Baseline characteristics of SOS individuals with LT and HT. Data are shown as mean ± SD, or proportions. Abbreviations: SOS, Swedish obese subjects; LT, low transaminase; HT, high transaminase; N, number; F, female; BMI, body mass index; HDL, high density lipoprotein; ALT, alanine transferase; AST, aspartate transferase. (DOC) [file pone.0060495.s003.doc]

**Table S1. Baseline characteristics of SOS individuals with LT and HT.**

|  | | **LT** | | | **HT** | | |
| --- | --- | --- | --- | --- | --- | --- | --- |
|  | **Control** | | **Surgery** | **P value** | **Control** | **Surgery** | **P value** |
| **N** | 1150 | | 957 |  | 645 | 818 |  |
| **Gender F, %** | 75 | | 75 | 0.92 | 67 | 70 | 0.40 |
| **Age, years** | 49±6 | | 47±6 | <0.001 | 49±6 | 47±6 | <0.001 |
| **Body weight, kg** | 113±16 | | 120±17 | <0.001 | 117±16 | 121±16 | <0.001 |
| **BMI, kg/m^2^** | 39.9±4.8 | | 42.4±4.7 | <0.001 | 40.7±4.6 | 42.5±4.3 | <0.001 |
| **Systolic blood pressure, mmHg** | 137±18 | | 145±19 | <0.001 | 139±18 | 146±19 | <0.001 |
| **Diastolic blood pressure, mmHg** | 84±11 | | 89±11 | <0.001 | 86±11 | 91±11 | <0.001 |
| **Blood glucose, mmol/L** | 4.8±1.7 | | 4.9±1.8 | 0.023 | 5.2±2.0 | 5.5±2.2 | 0.016 |
| **Total cholesterol, mmol/L** | 5.6±1.1 | | 5.8±1.1 | <0.001 | 5.6±1.1 | 5.9±1.3 | <0.001 |
| **Triglycerides, mmol/L** | 1.93±1.27 | | 2.08±1.13 | 0.005 | 2.11±1.45 | 2.44±1.96 | <0.001 |
| **HDL cholesterol, mmol/L** | 1.35±0.33 | | 1.36±0.32 | 0.69 | 1.32±0.31 | 1.32±0.32 | 0.86 |
| **ALT, U/L** | 23±7 | | 24±7 | 0.001 | 51±36 | 52±25 | 0.50 |
| **AST, U/l** | 19±4 | | 19±5 | 0.39 | 32±16 | 33±17 | 0.11 |
| **Alcohol consumption, g/day** | 3.0±4.0 | | 3.1±4.0 | 0.76 | 3.6±4.7 | 3.7±4.5 | 0.76 |
| **Diabetes, %** | 11 | | 15 | 0.002 | 26 | 29 | 0.431 |
| **Glucose-lower medication, %** | 5 | | 7 | 0.05 | 10 | 10 | 1.00 |
| **Lipid-lowering medication, %** | 2 | | 2 | 1.00 | 1 | 2 | 0.25 |
